# Structural and Pragmatic Language Impairments in Children Evaluated for Autism Spectrum Disorder (ASD)

**DOI:** 10.1007/s10803-020-04853-1

**Published:** 2021-01-30

**Authors:** Lise Reindal, Terje Nærland, Bernhard Weidle, Stian Lydersen, Ole A. Andreassen, Anne Mari Sund

**Affiliations:** 1 Department of Child and Adolescent Psychiatry, Møre og Romsdal Hospital Trust, Volda Hospital, Volda, Norway; 2 Department of Mental Health, Faculty of Medicine and Health Sciences, Regional Centre for Child and Youth Mental Health and Child Welfare, Norwegian University of Science and Technology, Trondheim, Norway; 3 NevSom, Department of Rare Disorders and Disabilities, Oslo University Hospital, Oslo, Norway; 4 K.G Jebsen Centre for Neurodevelopmental Disorders, University of Oslo, Oslo, Norway; 5 Department of Child and Adolescent Psychiatry, St. Olavs University Hospital, Trondheim, Norway; 6 NORMENT Centre, University of Oslo, Oslo, Norway; 7 Division of Mental Health and Addiction, Oslo University Hospital, Oslo, Norway

**Keywords:** Autism spectrum disorder, Language impairment, Structural language skills, Pragmatic language skills, Language milestones, Sex differences

## Abstract

Pragmatic language impairments are common in neurodevelopmental disorders, especially in autism spectrum disorder (ASD). The relationship between structural language skills and pragmatic competence in children with autistic symptoms, however, is largely unknown. We investigated this relationship based on the Children’s Communication Checklist-2 and early language delay among children (*N* = 177, 19% females) clinically evaluated for ASD, differentiated into ASD (*n* = 148) and non-ASD (*n* = 29). Structural language deficits were common and associated with reduced pragmatic competence in both groups. Pragmatic language impairments were most profound in children with ASD. Early language delay and structural language deficits were less common in females. Our findings suggest that assessment of structural language skills should be included in the evaluation of children with suspected ASD.

## Introduction

Neurodevelopmental disorders (NDDs) are characterized by impairments in one or more developmental domains, such as cognition, communication, social, and motor functioning, as a result of atypical brain development (American Psychiatric Association 2013; Moreno-De-Luca et al. 2013). Autism spectrum disorder (ASD) is a childhood onset NDD characterized by persistent deficits in social communication and interaction, as well as restricted, repetitive behavior and interests (American Psychiatric Association 2013; World Health Organization 1992). The common co-occurrence of different NDDs and the dimensional nature of their symptom profiles represent major challenges to the recognition, as well as the classification of these disorders (Baird and Norbury 2016). Many children with NDDs have language difficulties, particularly using language in social communication. In a clinical setting, however, language impairments are often unnoticed due to other, more prominent symptoms, and frequently remain undiagnosed (Cohen et al. 1998). Although a neglected area in current research, language impairment is suggested as an associated feature, independent from core ASD features in some aspects, with great importance for outcome in individuals on the autism spectrum (Happé and Frith 2020).

Within communication the *form, content* and *use* of language are all essential components. Language *form* (e.g. phonology, morphology, syntax) and *content* (semantics) represent *structural language skills*, while appropriate *use* of language in social or situational contexts represent *pragmatic language skills* (e.g. Geurts and Embrechts 2008; Baird and Norbury 2016). Language impairments reflect deficits in one or more of these skills, and vary depending on the individual’s age, intellectual level, as well as co-occurring difficulties in other developmental domains (Lord et al. 2018; Boucher 2012).

Impairments in pragmatic language are observed in a broad range of NDDs, including ASD (e.g. Bishop 1998; Norbury et al. 2004; Gilmour et al. 2004; Geurts and Embrechts 2008). Although not required for meeting diagnostic criteria, pragmatic impairments are a recognized feature of ASD regardless of language level or age (e.g., Baird and Norbury 2016; La Valle et al. 2020). Still, these impairments are often less emphasized than the social communication impairments inherent in the ASD diagnosis (Norbury 2014). Pragmatic skills require use of both the language and the social context to reach intended meaning. As such, they stand at the intersection of structural language and social skills (Volden et al. 2009). Norbury (2014) has argued that pragmatic language skills are closely associated with structural aspects of language, and not necessarily the same as social communication skills.

Although receiving less attention than pragmatic language deficits, structural language is also commonly affected in ASD. Preschool children with ASD show structural as well as pragmatic language impairments, resembling the language profile in children with specific language impairment (Geurts and Embrechts 2008; Boucher 2012). By school-age, however, structural deficits are reported to improve, while pragmatic language deficits become more prominent (Rapin and Dunn 2003; Geurts and Embrechts 2008). Moreover, an ASD-typical profile is reported to emerge in school-age, with articulation and syntax least affected, and comprehension, semantics and morphology most affected, as reviewed by Boucher (2012). Notably, children with ASD often evidence variability in skills across specific language domains, which appear to differentially relate to other aspects of functioning (Levinson et al. 2020). While previous work is limited and has disproportionately focused on the association between pragmatic language and social skill deficits, there are reports suggesting a link between structural language deficits and social skills in ASD, that is mediated by reduced pragmatic competence and may be at play for children without ASD as well (Volden et al. 2009; Levinson et al. 2020). Concomitant deficits in structural language may represent a potential target of intervention, separate from the social communication impairments characteristic of ASD. Therefore, investigating structural language skills and their potential influence on pragmatic competence in referred children with autistic symptoms is of importance.

ASD symptoms vary widely across individuals meeting diagnostic criteria for ASD and are also present in the general population to a minor degree (Constantino and Todd 2003, 2005; Posserud et al. 2006). For clinicians evaluating children with autistic symptoms, it may be challenging to disentangle core ASD symptoms from more specific language impairments that disturb social communication (Levy et al. 2010; Baird and Norbury 2016). It has been argued that the association between the different disorders affecting language and communication may best be understood dimensionally (Bishop and Norbury 2002; Bishop 2000). The individual differences in social communication and pragmatic language seen across various NDDs may then reflect a confluence of risk factors such as deficits in structural language, social and cognitive skills, with ASD at “the extreme end of the distribution” (Norbury 2014, p. 212), but without a disorder-specific profile. Investigating language impairments in a broader clinical population of children with autistic symptoms, beyond those receiving an ASD diagnosis, can offer an important complementary insight into the nature of these impairments and their extent in both ASD and non-ASD individuals.

The Children’s Communication Checklist (CCC-2) (Bishop 2011, 2003) is designed to identify structural and pragmatic language deficits that may be difficult to elicit in a test situation, and is to be completed by an adult who knows the child well (Norbury et al. 2004). Previous efforts to distinguish different NDDs based on their CCC-2 language profile have largely failed, but significant deficits in structural language in children with ASD compared to typically developing children are documented (Kuijper et al. 2017; Baixauli-Fortea et al. 2019; Geurts and Embrechts 2008). In addition pragmatic language impairments were evident in children across a range of NDDs, many of them had structural language deficits as well (Norbury et al. 2004; Geurts and Embrechts 2008). Recently, Baixauli-Fortea et al. (2019) reported an association between more advanced structural language skills and greater pragmatic competence in children with ASD, as measured by the CCC-2. On a continuum of communication impairment, ASD and specific language impairment are found on the opposite endpoints, with comparable structural language skills but more profound pragmatic impairments in children with ASD (Oi et al. 2017). However, design, measures, and comparison groups varied between these studies, limiting comparability and generalization of their results. Further, the ASD groups in many of these studies were relatively small. Thus, an unanswered question is whether pragmatic language impairment represents a dimensional trait that is associated with structural language deficits across the range of autistic symptoms.

While language milestones and current language skills have been important for distinction between ASD subtypes (e.g. World Health Organization 1992), they are not found to predict autistic symptom severity in children with ASD (Loucas et al. 2008; Kenworthy et al. 2012). Still, lasting individual differences in language skills seem to be established early, underscoring the importance of identifying lagging language skills early in life (Bornstein et al. 2018). Being a “late talker” (i.e. delayed attainment of first words and/or first word combinations) is considered a hallmark of specific language impairment (Conti-Ramsden and Durkin 2015), a condition characterized by structural language deficits. Delays in language milestones are also common in children later diagnosed with ASD, and represent early signs of the condition, although with low specificity (Tager-Flusberg 2016). Measured by a sentence repetition task, retrospectively reported language milestones were predictive of later structural language skills in children with ASD (Kenworthy et al. 2012). Whether milestone data can be useful markers of later language performance also across the broader range of autistic symptoms, as measured by the CCC-2, remains to be resolved.

Females demonstrated better pragmatic language skills on the CCC (Ketelaars et al. 2010; Geurts et al. 2009) and its successor, the CCC-2 (Ash et al. 2017) in community-based samples. However, no significant sex differences were found in a Norwegian normative sample (Hollund-Møllerhaug 2010). Regarding ASD, females may present with a different profile of symptoms than males, and therefore be under- or misdiagnosed, or diagnosed with delay (Green et al. 2019; Kreiser and White 2014; Van Wijngaarden-Cremers et al. 2014). At present, studies exploring potential sex differences in language characteristics within the broader group of children with autistic symptoms are lacking.

While originally autism was conceptualized as distinct from typical development, a more recent conception is the dimensional, with ASD as a spectrum of manifestations and no natural cut-off point between high autism traits and ASD (Happé and Frith 2020). The same authors argue that an unintended consequence of focusing on ‘pure’ autism has been the neglect of language impairment in recent research (Happé and Frith 2020). By including a large group of children evaluated for ASD by specialist health services, some not fulfilling the criteria for such a diagnosis (non-ASD), we aimed to use a dimensional approach and study language impairment across the broader range of autistic symptoms. Four specific objectives were addressed:

(i) To investigate the extent of language deficits based on the CCC-2 (composite and subscale scores) and parents retrospective report of early language delay.
(ii) To investigate whether current structural language skills are associated with pragmatic competence (as measured by CCC-2 composite scores).
(iii) To explore whether parent reported early language delay predict current language and social skills as measured by CCC-2 composite scores and Social Responsiveness Scale (SRS) total score.
(iv) To explore potential sex differences in language characteristics.

## Methods

### Study Design

The present study is part of BUPgen, an ongoing large multi-site study of neurodevelopmental disorders in Norway, in which children are eligible for enrollment if a suspicion of ASD has been raised by local or specialist health services. This study involved analyses of data collected and processed by April 2019. Data are collected from two types of sites: (1) child habilitation services and (2) child and adolescent mental health services, i.e. public specialist health services receiving referrals for assessment of ASD. After written, informed consent to participate, information from patients’ records was extracted by clinicians, following standard procedures.

### Participants

Participants were eligible if information on age (4–18 years) at inclusion, diagnostic classification as either ASD or non-ASD, and results from assessment with the Children’s Communication Checklist Second Edition (CCC-2) was available. In total, *N* = 177 children were included, born between 1994 and 2012, with a mean age at inclusion of 12.3 years (standard deviation (*SD*) = 3.3). As the CCC-2 is only completed when the child can speak in at least simple sentences, all participants were verbal. Children were not excluded from participation if they were bilingual speakers of Norwegian (*n* = 6), if they had histories of impaired hearing (*n* = 14) or receiving services from a speech therapist (*n* = 21). Data included results from present and previous clinical assessments, parent-reported history and supplementary parent-reported measures.

Participants consisted of 148 children (83.6%) with a clinical diagnosis of any ASD according to ICD-10 (F84x) and 29 children (16.4%) with suspected ASD, but no clinical ASD diagnosis (non-ASD). Common ASD subtypes included Asperger syndrome (AS) (80/148, 54.1%), Pervasive developmental disorder not otherwise specified (45/148, 30.4%), Childhood autism (14/148, 9.5%) and Atypical autism (7/148, 4.7%), whereas the majority of non-ASD children had one or more NDDs (21/26, 80.8%). Other NDDs were grouped according to ICD-10 codes into the following categories: intellectual disability (F70-79), attention-deficit/hyperactivity disorder (ADHD) (F90), communication disorder (F80), specific learning disorder (F81 and F83), motor disorder (F82 and F95), other NDD (F88, F89 and F94). The presence of epilepsy or cerebral palsy was also registered and included in the total number of NDDs.

### Assessments

#### Diagnoses

All diagnoses were assigned by Norwegian specialist health services, using the *International Statistical Classification of Diseases, 10th Revision* (ICD-10) criteria (World Health Organization 1992). The majority of ASD (121/148, 81.8%) and non-ASD individuals (20/28, 71.4%) had completed the Autism Diagnostic Observation Schedule (ADOS) (Lord et al. 1999), or the Autism Diagnostic Interview-Revised (ADI-R) (Rutter et al. 2003b), or both as part of the clinical evaluation. In cases where ADI-R had not been administered, the Social Communication Questionnaire (SCQ), Lifetime form (Rutter et al. 2003a) was completed at inclusion—if not performed earlier.

#### Early Language Development

A clinician rated medical history form was obtained for all participants at inclusion, which inquired whether the child had attained one spoken word at 1 years’ age, and whether the child had attained its first phrase (a spoken two-word combination) at 2 years’ age. This assessment was completed based on the child’s medical record supplemented by parent report, asking parents to retrospectively recall this information. Among children with ASD and normal range cognitive abilities, Kenworthy et al. (2012) found attainment of first phrase speech by 2 years’ (24 months) age to be a useful marker for distinguishing later language trajectories. For simplicity, therefore, not having attained first phrase at 2 years’ age was used as a proxy for early language delay in the present study.

#### Current Language and Communication Skills

The Children’s Communication Checklist Second Edition (CCC-2) (Bishop 2003; Norwegian version: Bishop 2011) is a caregiver reported measure that identifies children with language impairment in both clinical (Norbury et al. 2004) and community contexts (Ketelaars et al. 2009). The CCC-2 consists of 70 items grouped into 10 subscales that measure different aspects of communication: language structure (A: speech, B: syntax, C: semantics, D: coherence), pragmatic language skills (E: inappropriate initiation, F: stereotyped language, G: use of context, H: nonverbal communication), and two scales measuring social aspects (I: social relations and J: interests). The raw scores are converted into scaled scores with a mean of 10 and an SD of 3 based on Norwegian norms, that can also be converted into percentiles for each subscale. The Norwegian version of the CCC-2 has satisfactory internal consistency (Cronbach alpha ranging from 0.73 to 0.89) and inter-rater reliability (Spearman’s rho ranging from 0.44 to 0.76) (Helland et al. 2009). The checklist does not provide a categorical diagnosis, but subscales may be combined as composites. The General Communication Composite (GCC) is an overall measure of communication skills, derived by adding the scaled scores of the subscales A-H. In scaled scores a high score indicates language strength and a low score language deficit. A GCC below 55 is considered the cut-off for distinguishing children with clinically significant language impairment from typically developing (TD) children (Bishop 2011). We calculated the Structural Language Score, obtained by adding together the scores on the structural scales (A-D) and the General Pragmatics Score by adding together the scores on the four pragmatic scales (E–H), without the two social nonlinguistic scales (I, J). This specific grouping has been used in other studies (Baixauli-Fortea et al. 2019; Kuijper et al. 2017). Contrary to these, we report scaled scores (see Appendix for further discussion).

#### Current Social Impairment

The Social Responsiveness Scale (SRS) (Constantino and Gruber 2005) is a 65-item caregiver questionnaire that examines a child’s ability to engage in reciprocal social interaction. The SRS total score is a valid quantitative measure of autistic social impairment or traits, with higher scores indicating greater severity (Constantino et al. 2003). Previous reports indicate excellent internal consistency of the SRS, with a Cronbach alpha coefficient of .97 (Constantino and Gruber 2005). In the present study, we applied SRS raw total as a dimensional trait variable reflecting current (last 6 months) level of social impairment.

#### Cognitive Abilities

Cognitive function was assessed using results from age-appropriate Wechsler scales (*n* = 169): the Wechsler Preschool and Primary Scale of Intelligence (Wechsler 2012; 12.4%), Wechsler Intelligence Scale for Children (Wechsler 2003; 81.7%), Wechsler Abbreviated Scale of Intelligence (Wechsler 1999; 3.0%), and Wechsler Adult Intelligence Scale (Wechsler 2008; 3.0%). These assessments yield standard scores for nonverbal IQ (NVIQ), verbal IQ, and full-scale IQ. Mean age at assessment of cognitive abilities in the present sample (*n* = 168) was 10.0 (*SD* = 3.4) years. To minimize the effect of language in measuring cognitive abilities, we used NVIQ as a trait variable, reflecting severity of cognitive impairment.

### Statistical Analyses

Descriptive statistics are presented as *n* (%) and mean (*SD*). First, we report the extent of language deficits by the mean (SD) for the CCC-2 composite and subscale scores. We also assessed the proportion of children with scores below the chosen cut-off to indicate significant deficits (i.e. GCC < 55 or subscale score ≤ 5th percentile compared to the Norwegian norms, respectively). Second, we investigated whether current structural language skills were associated with pragmatic competence across the whole sample by performing a linear regression analysis with the General Pragmatics Score as dependent variable. The analysis was carried out unadjusted and adjusted for potential confounders, one at a time, and simultaneously. Potential confounding factors included were NVIQ, age at inclusion, and sex. Third, we divided the sample into two groups based on early language delay (i.e. not having attained first phrase at 2 years’ age) and compared current language and social skills between these groups. We used independent sample *t*-test and Pearson’s chi-squared for between-group comparisons. Mean CCC-2 composite scores were compared using linear regression, adjusting for cognitive ability (NVIQ) and age at inclusion (years). To compare proportions, we computed the Newcombe hybrid score confidence interval as recommended by Fagerland et al. (2015) using Stata 16, and the unconditional z-pooled test as recommended by Lydersen et al. (2012) using StatXact 11. Finally, to explore possible sex differences, group comparisons were repeated for males and females within the whole sample. Possible sex differences in the association between structural and pragmatic language skills were explored in a subsequent regression analysis including an interaction term between sex and the Structural Language Score.

We report available case analyses with the corresponding number of missing cases where appropriate. Following the example of Geurts and Embrechts (2008) we conducted these analyses with (*n* = 177) and without (*n* = 153) the inclusion of participants with invalid consistency check on the CCC-2. As the results in general were the same, the values in tables and figures include all children (*n* = 177). Two-sided *p* values < 0.05 were regarded as statistically significant. In order to protect against type I error due to multiple hypotheses, however, we recommend *p*-values between .01 and.05 to be interpreted with caution. Except otherwise noted, we used SPSS 26 for statistical analyses.

## Results

### Sample Characteristics

The main sample (*N* = 177) included 143 males (80.8%) with a male to female ratio of approximately 4:1 (Table 1). Most children (148/177, 83.6%) had an ASD diagnosis. The majority of children that did not meet the criteria for an ASD diagnosis (non-ASD) were diagnosed with one or more NDDs, mainly ADHD (17/27, 63.0%), specific learning disorders (6/27, 22.2%), and motor disorders (5/27, 18.5%). Co-occurrent ADHD was equally frequent among children with ASD (86/145, 59.3%) and did not differ between groups. Within the whole sample, participating females (*n* = 34) were older at inclusion compared with males (13.5 (*SD* = 3.2) versus 12.0 (*SD* = 3.3) years), and females with ASD had received their diagnosis later (13.6 (*SD* = 2.8) versus 11.0 (*SD* = 3.3) years among ASD males).

**Table 1:** Participant characteristics (*N* = 177)

	*n*	(%)	Range	Mean (SD)
Male sex	143	80.8		
Age (years) at inclusion	177		4–18	12.3 (3.3)
Age (years) at ASD diagnosis	144			11.5 (3.3)
Current social impairment (SRS total)	162		9–153	83.4 (29.8)
Age (years) at cognitive testing	168		4–18	10.0 (3.4)
Nonverbal IQ	161		59–142	102.5 (18.4)
Verbal IQ	163		53–124	91.4 (16.9)
Early language milestones				
One word 1 year (no)	32	22.2		
Two words 2 year (no)	38	27.1		
Diagnoses				
ASD (F84)	148	83.6		
Intellectual Disability (F70-79)	8	4.6		
ADHD (F90)	103	59.9		
Communication disorder (F80)	7	4.1		
Specific learning disorder (F81 + F83)	18	10.5		
Motor disorders (F82 + F95)	27	15.7		
Epilepsy	10	5.6		
Cerebral Palsy	2	1.1		
Other NDD (F94)	1	0.6		
No of NDDs				
0	5	2.8		
1	58	32.8		
≥ 2	106	59.9		
Ethnicity				
European (Caucasian)	157	88.7		

Data are expressed as *n* (%) or mean (SD). The denominator for the reported proportions in this table excludes those with missing data. IQ was obtained from various age-appropriate standardized tests

*ASD* autism spectrum disorder, *SRS* Social Responsiveness Scale, *NDD* neurodevelopmental disorder

Mean age at ASD diagnosis was 11.5 years (*SD* = 3.3). Children with ASD had higher mean scores on diagnostic measures as well as the measure of current social skills (SRS) compared with non-ASD (*p* < .01, all). Non-ASD individuals were younger at inclusion (11.0 years (*SD* = 3.7) versus 12.5 years (*SD* = 3.2) in the ASD group). Mean age at assessment of cognitive abilities and at administration of ADI-R, however, did not differ between the groups. Lastly, mean values of nonverbal and verbal cognitive abilities were in the normal range and without significant group differences (see Appendix for details on characteristics in both groups).

### Extent of Language Deficits Across the Range of Autistic Symptoms

Most children (144/177, 81%) were classified as language impaired, by the CCC-2 (GCC < 55) (Table 2). In general, pragmatic language deficits were more common than structural deficits. Among the structural language skills, ‘syntax’ was least affected. Still, 27% of children had significant deficits on this subscale (≤ 5th percentile). Moreover, 66% had significant deficits on the ‘coherence’ subscale, which was the most affected structural scale. For all subscales measuring pragmatic aspects of language, more than half of the sample presented with significant deficits. The most affected pragmatic skill in both groups was nonverbal communication. However, in children with ASD, the deficits on the ‘nonverbal communication’ subscale were more profound (4.2 (*SD* = 2.7)) than in the non-ASD group (5.6 (*SD* = 2.9); *p* = .01). The ASD group also performed worse on the General Pragmatics Score compared to the non-ASD group (16.3 (*SD* = 9.2) versus 21.1 (*SD* = 11.3); *p* = .01). Both groups performed equally on the GCC and the Structural Language Score. Adjusting for NVIQ and age at inclusion did not alter these findings substantially. Notably, language impairment was not universal. Within the whole sample, 33 children (19%) did not have any language impairment as measured by the CCC-2. A minority (38/140, 27%) had reported early language delay, i.e. not having attained first phrase at 2 years’ age (Table 1). Analyses comparing characteristics between individuals with (*n* = 153) and without (*n* = 22) valid consistency check on the CCC-2 are presented in the Appendix.

**Table 2:** CCC-2 subscale and composite scores (a high score indicates better language ability): means, standard deviations, proportion below ‘cut-off’ indicating significant deficits for the whole sample, the ASD and the non-ASD group

	Whole sample			ASD			Non-ASD		
	*N* = 177			*n* = 148			*n* = 29		
	Mean	SD	Below ‘cut-off’ (%)	Mean	SD	Below ‘cut-off’ (%)	Mean	SD	Below ‘cut-off’ (%)
CCC-2 subscale scores			≤ 5 percentile*			≤ 5 percentile*			≤ 5 percentile*
A. Speech	6.5	3.9	35.0	6.6	4.0	35.1	5.9	3.6	34.5
B. Syntax	6.5	3.6	27.1	6.6	3.6	27.0	6.4	3.6	27.6
C. Semantics	5.0	3.1	34.5	4.8	3.0	35.8	5.9	3.4	27.6
D. Coherence	4.0	3.0	66.1	3.9	2.9	67.6	4.7	3.5	58.6
E. Inappropriate initiation	4.5	2.6	55.4	4.3	2.4	58.1	5.6	3.2	41.3
F. Stereotyped language	4.8	3.0	50.8	4.6	3.0	52.0	5.7	3.0	44.8
G. Use of context	3.4	3.0	57.1	3.2	2.9	60.1	4.3	3.4	41.4
H. Nonverbal communication	4.4	2.8	71.8	4.2	2.7	75.7	5.6	2.9	51.7
I. Social relations	3.3	2.9	73.4	3.0	2.6	77.7	5.1	3.6	51.7
J. Interests	3.3	2.3	60.5	3.1	2.2	66.2	4.7	2.5	31.0
CCC-2 composite scores			GCC < 55			GCC < 55			GCC < 55
GCC (sum scales A–H)	39.1	17.9	81.4	38.1	17.2	83.8	44.2	20.9	69.0
Structural Language Score (sum scales A–D)	22.0	10.3	n.a	21.8	10.0	n.a	22.9	11.9	n.a
General Pragmatics Score (sum scales E–H)	17.1	9.7	n.a	16.3	9.2	n.a	21.1	11.3	n.a

*ASD* autism spectrum disorder, *CCC-2* Children’s Communication Checklist Second Edition, *SD* standard deviation, *n.a.* not applicable

*Proportion (%) of individuals with subscale score at or below the 5th percentile compared to Norwegian norms

### The Relationship Between Current Structural Language Skills and Pragmatic Competence

**Fig. 1:**
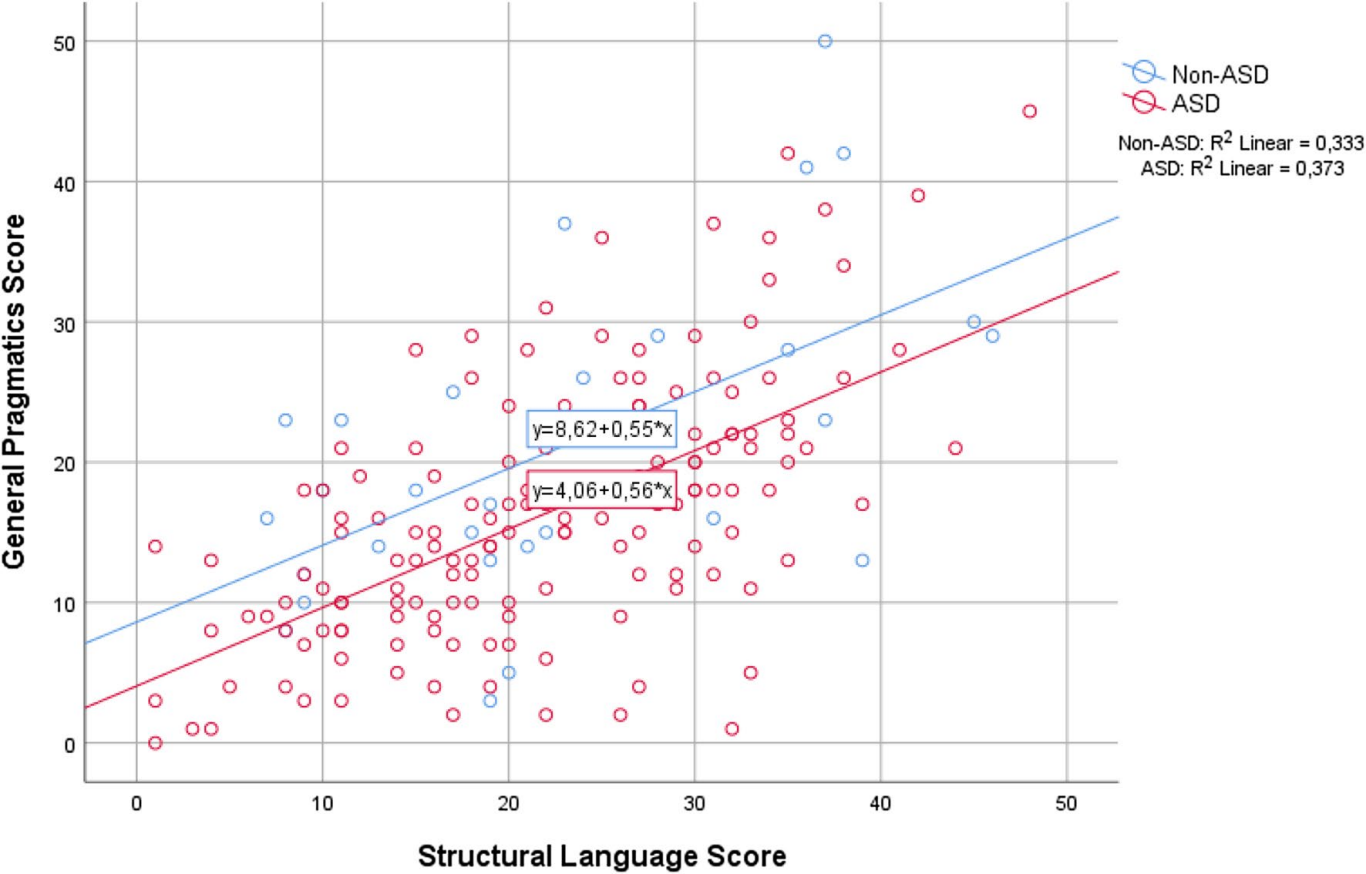
Distribution of Structural Language and General Pragmatics composite scores across the study sample (*N* = 177), and their linear associations in the group with and without diagnosed autism spectrum disorder (ASD; *n* = 148 and non-ASD; *n* = 29)

The Structural Language Score was strongly associated with the General Pragmatics Score with a regression coefficient 0.56 (CI 0.45 to 0.68), *p* < .001, and explained 35.9% of the variance in the General Pragmatics Score. After adjustment for potentially confounding variables, the association remained substantially unchanged (Table 3). The potential influence of diagnostic group on the observed association was also explored. As illustrated in Fig. 1, current structural and pragmatic language skills, as measured by the CCC-2, were highly correlated regardless of diagnostic group.

**Table 3:** Linear regression with General Pragmatics Score as dependent variable and Structural Language Score as primary covariate (scaled scores)

	Correlation coefficient			*p*
	*n*	B	95% CI	
*Unadjusted*				
Structural Language Score	177	.56	(.45 to .68)	< .001
*Adjusted separately for*				
Sex (female)	177	.57	(.45 to .68)	< .001
Age (years)	177	.57	(.46 to .68)	< .001
Nonverbal IQ	161	.60	(.48 to .72)	< .001
*Adjusted for all*	161	.60	(.48 to .72)	< .001

Results based on available case analysis of the main sample

*B* unstandardized regression coefficient, *CI* confidence interval, *p p*-value

### Early Language Delay and Current Language and Social Skills

**Fig. 2:**
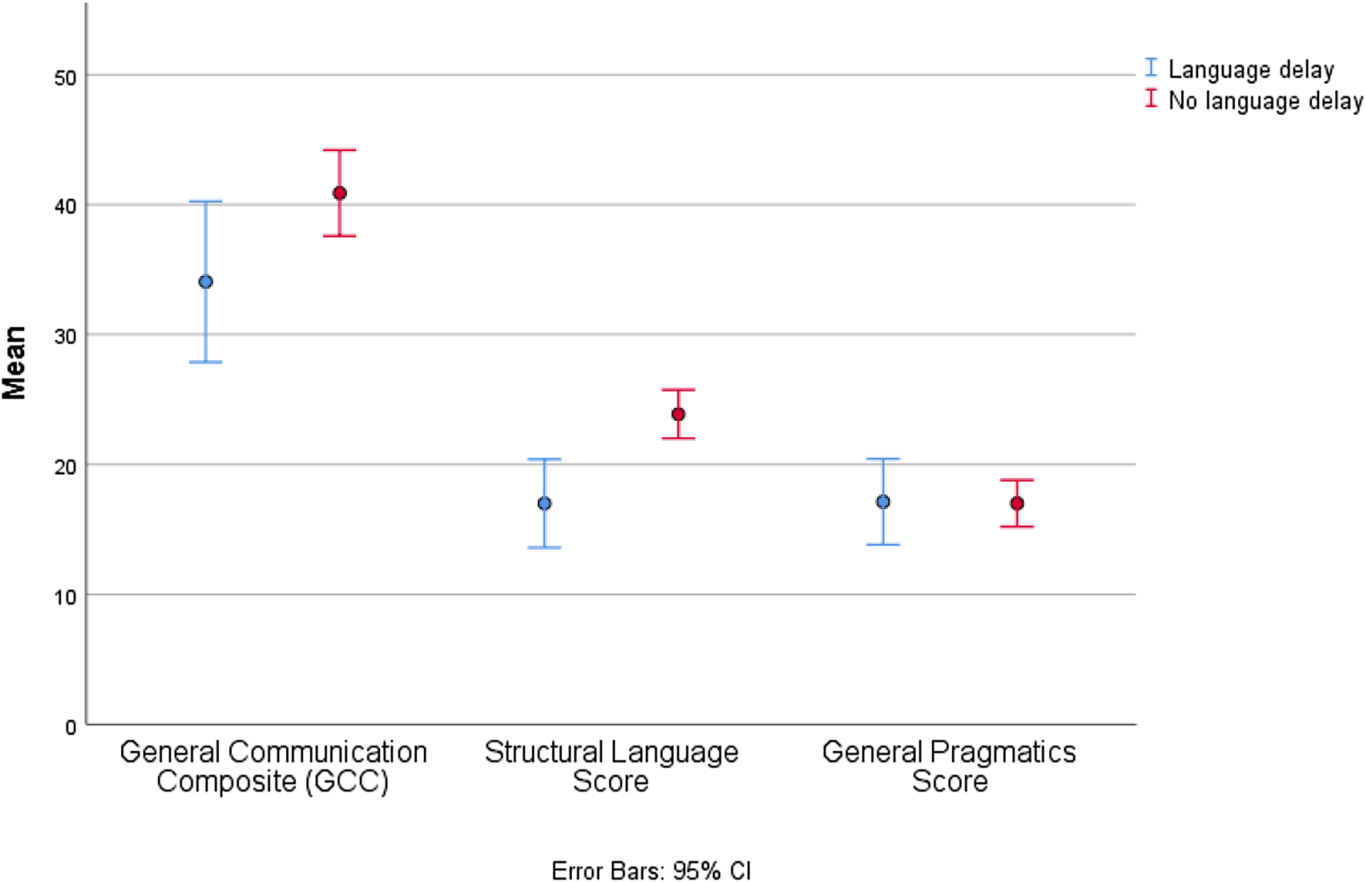
Clustered error bar mean of CCC-2 composite scores of children (*n* = 140) with parent report on early language delay, separated into children with (*n* = 38) and without (*n* = 102) early language delay (i.e. not having attained first phrase at 2 years’ age). Means and 95% CI

Within the whole sample, the 38 children with reported language delay performed worse on current measures of general communication (GCC; 34.1 (*SD* = 18.8) versus 40.9 (*SD* = 16.8); *p* = .04) and structural language skills (Structural Language Score; 17.0 (SD = 10.4) versus 23.9 (SD = 9.5); *p* < .001) compared with the 102 children without language delay. No significant difference was found regarding pragmatic skills (Fig. 2). Adjusting for NVIQ and age at inclusion did not alter the findings substantially, except that the difference in GCC no longer was significant (*p* = .20). Children in the language delayed group also performed worse on measures of verbal IQ (80.8 (*SD* = 16.2) versus 94.9 (*SD* = 15.1), respectively; *p* < .001), while no difference was found regarding current social skills (SRS total raw score), when compared with the group without language delay. Children receiving an ASD diagnosis were diagnosed earlier if they had early language delay (10.1 years (*SD* = 4.0) versus 11.9 years (*SD* = 2.9); *p* = .03).

### Sex Differences

**Fig. 3:**
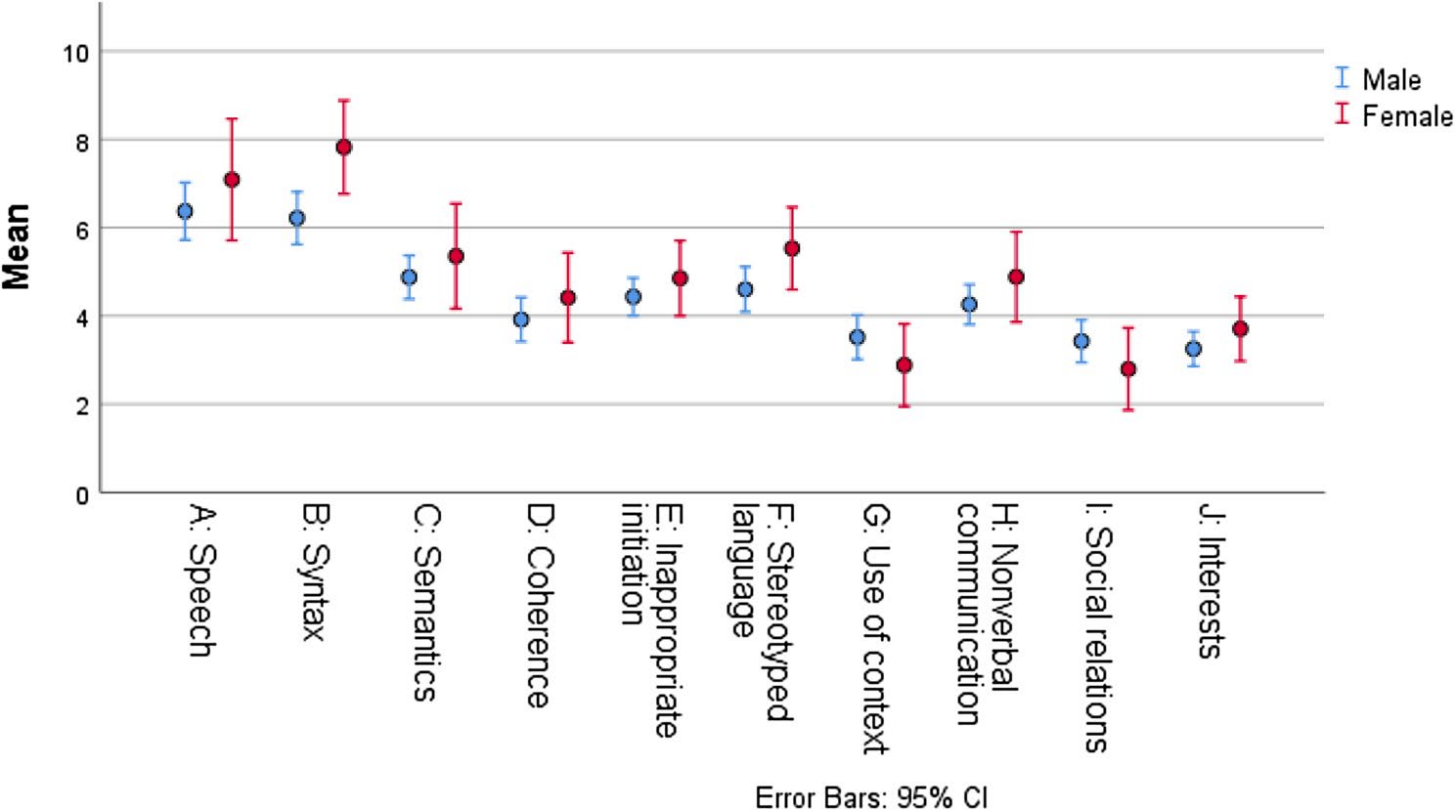
Clustered error bar mean of CCC-2 subscale scores in the total study sample (*N* = 177), by sex. Means and 95% CI

The majority of both males (117/143, 82%) and females (27/34, 79%) was identified as language impaired (GCC < 55), and the overall extent and profile of language impairments, as measured by the CCC-2 composite scores, did not differ by sex (unadjusted and adjusted for potential confounders) (Table 4). Generally, females had higher mean scores (indicating better performance) on most subscales, although reaching statistical significance only for the ‘syntax’ subscale (*p* = .02) (Fig. 3). There was no significant interaction between sex and Structural Language Score on the General Pragmatics Score. Females, however, performed better than males on measures of verbal IQ (96.5 (*SD* = 16.2) versus 90.1 (*SD* = 16.8), respectively, *p* = .05). Only one female (1/27, 4%) was reported with language delay compared with males (37/113, 33%), *p* = .003.

**Table 4:** Participant and language characteristics by sex (*N* = 177)

	Males (*n* = 143)			Females (*n* = 34)			Difference		
	*n*	(%)	Mean (SD)	*n*	(%)	Mean (SD)	Estimate	95% CI	*p*
ASD	119	83.2		29	85.3		− 2.1	(− 12.9 to 14.2)	.79
Age (years) at inclusion	143		12.0 (3.3)	34		13.5 (3.2)	− 1.5	(− 2.8 to − .3)	.02
Age (years) at ASD diagnosis	118		11.0 (3.2)	26		13.6 (2.8)	− 2.6	(− 4.0 to − 1.2)	< .001
Age (years) at cognitive testing	135		9.8 (3.4)	33		10.9 (3.2)	− 1.1	(− 2.4 to .15)	.08
Nonverbal IQ	128		102.7 (19.6)	33		101.8 (12.9)	1.0	(− 4.7 to 6.6)	.74
Verbal IQ	130		90.1 (16.8)	33		96.5 (16.2)	− 6.4	(− 12.8 to .1)	.05
Early language milestones									
One word 1 year (no)	30	25.6		2	7.4		18.3	(.8 to 28.4)	.04
Two words 2 year (no)	37	32.7		1	3.7		29.0	(12.4 to 38.6)	.003
Language impaired (GCC < 55) CCC-2 composite scores	117	81.8		27	79.4		2.4	(− 10.1 to 19.5)	.7 7
GCC (sum scales A–H)	143		38.2 (18.4)	34		42.9 (15.7)	− 4.7	(− 11.4 to 2.1)	.17
Structural Language Score (sum scales A–D)	143		21.4 (10.5)	34		24.7 (9.0)	− 3.3	(− 7.2 to .6)	.09
General Pragmatics Score (sum scales E–H)	143		16.8 (10.0)	34		18.2	− 1.3	(− 5.0 to 2.3)	.47

Data are expressed as *n* (%) or mean (SD). The denominator for the reported proportions in this table excludes those with missing data. IQ was obtained from various age-appropriate standardized tests

*ASD* autism spectrum disorder, *NDD* neurodevelopmental disorder

## Discussion

In this study of language characteristics in a sample of children evaluated for ASD by specialist health services we found that the majority had language impairment, i.e. general communication skills below the CCC-2 cut-off (GCC < 55). Structural language deficits were common and strongly associated with pragmatic competence across the whole sample. Pragmatic language impairments were most profound in children with ASD. Early language delay was more common among males and associated with structural language deficits, whereas pragmatic language and social skills did not differ significantly among children with and without language delay. Our findings support that pragmatic language impairment as a dimensional symptom profile probably reflect a confluence of risk factors, among them structural language deficits. Further, they support that early language delay is associated with later language abilities that are distinct from autistic symptoms. Lastly, we contribute to recent reports that females with ASD may be recognized and diagnosed later than males probably due to stronger verbal skills and a reduced rate of early language delay.

### Structural and Pragmatic Language Deficits Across the Range of Autistic Symptoms

Existing research on language impairment often overlooks differences in autism severity (Levinson et al. 2020). As a result, little is known about how distinct language skills may present differently across the autism spectrum. In the present study, we applied a dimensional approach and studied language skills in a sample of children evaluated for ASD, with and without ASD diagnoses. We found a large extent of language impairment across the whole sample, as measured by the CCC-2 (GCC < 55), that did not differ significantly between children diagnosed with ASD (84%) and children not fulfilling the diagnostic criteria (non-ASD; 69%). The observed extent of language impairment is comparable to previous findings among children with Asperger syndrome and children with ADHD (Helland et al. 2012), both of which were common diagnoses in the present sample.

Although both structural and pragmatic language skills were widely distributed across both groups, pragmatic aspects (the *use*) of language were most affected. This is in line with previous results among school-aged children with ASD (Geurts and Embrechts 2008; Boucher 2012). As expected, the ASD and non-ASD group differed significantly on the subscales that map social deficits characteristic of ASD (‘social relations’ and ‘interests’, *p* = .004 and *p* = .001, respectively). Although our non-ASD group was small (*n* = 29), significant pragmatic deficits were found compared to Norwegian norms, albeit less profound than in the ASD group. These results support the concept of pragmatic language impairment as a dimensional symptom profile present across a range of NDDs, with ASD “at the extreme end”, as suggested by Norbury (2014, p. 212). Pragmatic skills include a child’s ability to initiate and maintain a mutual conversation, to flexibly adapt the use of language to the social context and resolve ambiguities, as well as non-verbal aspects of communication. Our findings coincide with previous studies using the CCC-2 that have reported more profound pragmatic impairments among children with ASD compared to typically developing children (e.g. Geurts and Embrechts 2008; Oi et al. 2017; Helland et al. 2012), but also compared to children with other NDDs, such as specific language impairment (Oi et al. 2017; Geurts and Embrechts 2008; Norbury et al. 2004), and ADHD (Geurts and Embrechts 2008; Kuijper et al. 2017; Helland et al. 2012). With the exception of Oi et al. (2017) who investigated whether aspects of communicative impairment were continuously distributed in a population-based sample, these studies compared categorically defined clinical groups, which were also considerably smaller than the ASD group in the present sample. Applying a dimensional approach, we extend their findings to a larger clinical population of children with autistic symptoms.

Together, the CCC-2 structural scales (‘speech’, ‘syntax’, ‘semantics’, ‘coherence’) assess language functions apart from pragmatics that are commonly affected in children with specific language impairment (Norbury et al. 2004), including vocabulary and articulatory issues. By combining these subscales, we were able to assess structural aspects of the child’s language, as assessed by their caregivers. This includes the ability to apply rules for producing and combining speech sounds and combinations of words to form phrases and sentences, as well as the ability to understand and use the meaning of words and sentences, and to use a coherent language. We found that structural language deficits are common (compared to Norwegian norms) in children evaluated for suspected ASD, with deficits in ‘syntax’ being relatively infrequent, and ‘coherence’ being the most affected subscale. The overall extent and profile of structural language deficits did not differ between children with and without an ASD diagnosis, and is consistent with the language profile reported from previous studies in school-aged children with ASD, as reviewed by Boucher (2012). Further, the observed extent of deficits is comparable to previous studies in school-aged children with ASD (Helland et al. 2012; Kuijper et al. 2017; Baixauli-Fortea et al. 2019). Clinicians and researchers have long been aware of the high comorbidity between ASD and other NDDs (Lord et al. 2018), as well as their potential impact on specific aspects of language and communication. Still, studies on language skills in ASD rarely provide information on these comorbid diagnoses (Levinson et al. 2020). In the present study the proportion of children diagnosed with (co-occurrent) ADHD was high in both the ASD and the non-ASD group. Our finding that structural language skills were equally impaired in both groups are consistent with previous reports that children with ASD and ADHD are not possible to distinguish from each other on CCC-2 structural scales, while on pragmatic scales they can (Kuijper et al. 2017; Geurts and Embrechts 2008; Helland et al. 2012).

Co-occurring language impairment may influence the presentation of ASD symptoms, as well as the functional impairment of the child. Therefore, assessment of language skills is recommended as part of the diagnostic evaluation for ASD (Hyman et al. 2020). In line with Kjelgaard and Tager-Flusberg (2001) we report considerable heterogeneity in the language skills of children with ASD, but a somewhat smaller proportion of children with no language impairment. In our sample, only 16% (24/148) of children with ASD had no language impairment (GCC > 55). Suren et al. (2019a) reviewed patients records obtained from the specialist health service for 503 children with ASD in Norway, finding that the assessments largely were conducted in accordance with local guidelines. Notably, however, only a minority of children in their study underwent a formal assessment of language as part of their clinical evaluation (33%). Although the present sample consist of children who underwent an assessment using the CCC-2, our findings underscore that structural language deficits are frequent across the range of autistic symptoms and important to assess also in verbal children evaluated for ASD.

### High Correlation Between Current Structural and Pragmatic Language Skills

Previous work that has examined the relationship between specific language domains and other aspects of functioning has largely focused on the association between pragmatic language and social skills deficits in ASD. The expression of pragmatic competence often relies on verbal skills. As such, the close relationship between structural and pragmatic language skills observed in the present sample is expected, and consistent with previous reports of an association between structural and pragmatic language skills in children with specific language impairment (Ketelaars et al. 2009) as well as children with ASD (Volden et al. 2009; Baixauli-Fortea et al. 2019; Levinson et al. 2020). We replicate and extend their findings to a large group of children with a broad range of autistic symptoms. By investigating this relationship in a broader clinical population, we found that structural and pragmatic language skills, as measured by the CCC-2, were highly correlated regardless of diagnostic group. This suggests that the close relationship between structural and pragmatic language skills is present not only in children with ASD, but also in children with autistic symptoms seen across various NDDs. Further, our finding that pragmatic competence was statistically not solely explained by structural language skills is compatible with the notion that pragmatic language impairments might reflect a confluence of risk factors, among them deficits in structural language (Norbury 2014). Volden et al. (2009) not only reported structural language skills to predict performance on a standardized measure of pragmatic language in youth with ASD, but also that pragmatic language in turn uniquely predicted social skills. Taken together, these and the present findings suggest that although mediated by pragmatic language, structural language skills may influence social skills, and demonstrate the necessity of examining language skill domains separately when evaluating children with suspected ASD.

Notably, both composites used in the present analyses include various aspects of structural and pragmatic skills. The Structural Language Score include both *form* (‘speech’ and ‘syntax’) and *content* (‘semantics’ and ‘coherence’) skills, that may also tap into vocabulary knowledge and discourse. Although a strong correlation was found, it is likely that some aspects of structural language bear a stronger significance on pragmatic competence than others. Further, some aspects of pragmatic competence may be stronger related to structural language than others. In a CCC-2 validation study, Norbury et al. (2004) reported no group differences between children with specific language impairment and groups thought to have more severe pragmatic difficulties on the ‘stereotyped language’ and ‘use of context’ subscales, suggesting that structural language difficulties may influence ratings on these subscales. For instance, a child with limited expressive skills may rely on a few phrases that might appear stereotyped. Moreover, children with specific language impairment demonstrated strengths in ‘nonverbal communication’, suggesting that their structural deficits did not impact this aspect of pragmatic competence (Norbury et al. 2004).

The profile of language impairments in children with ASD is reported to change with pragmatic impairments becoming more prominent relative to structural deficits by school-age (Rapin and Dunn 2003; Geurts and Embrechts 2008). Such changes may be related to maturity, interventions, the interplay of developmental risk factors to cause more profound impairments over time (Geurts and Embrechts 2008), as well the pervasiveness of pragmatic language impairment becoming more apparent with increasing demands. The present sample mainly comprised school-aged children, and the cross-sectional design does not allow conclusions regarding language trajectories. Importantly, however, we report structural language deficits to be common in school-age children evaluated for suspected ASD, and to be strongly associated with pragmatic competence across the range of autistic symptoms.

### Early Language Delay and Current Language and Social Impairment

Deficits in pragmatic language and social communication may not become fully manifest until demands exceed limited capacity (Baird and Norbury 2016). As young children with clear developmental disabilities are likely to be referred earlier for specialist assessment than those without, it has been cautioned against overlooking young children with ASD and no language delay (Lord et al. 2018). As expected in a sample of verbal children, the proportion of children with language delay in the present study was relatively low, but comparable to findings from the Norwegian MoBa cohort (Suren et al. 2019b). While children with language delay had more structural language deficits compared to children without language delay, they did not differ in pragmatic language and social skills. In an earlier study Kenworthy et al. (2012) reported age of first phrases among verbal children with ASD to predict later structural language, but not other social communicative impairments characteristic of ASD. Moreover Loucas et al. (2008) found phrase speech to be acquired significantly later in ASD children with co-occurrent language impairment compared to those without, while current autistic symptoms and pragmatic language impairment did not differ. Although caution when interpreting retrospectively reported language milestone data is recommended (Hus et al. 2011; Ozonoff et al. 2018), these and the present findings suggest that early language delay represents an important predictor of later language ability that is distinct from autism symptoms. Further, they lend support to the recent revisions of *the Diagnostic and Statistical Manual of Mental Disorders* (DSM-5) and *the International classification of diseases* (ICD-11), where delayed or impaired language is no longer included as a core symptom of ASD, but should be specified as co-occurrent language impairment (American Psychiatric Association 2013; World Health Organization 2018).

### Sex-Based Differences in Language Profile

Assessing male and female language profiles separately may contribute to a better understanding of the female ASD phenotype. Consistent with findings in clinically-referred children with ASD (Solomon et al. 2012) we found no significant sex differences on the CCC-2 composite scores. We did, however, find that females presented with a relative strength in their structural language skills, performing better than males on the ‘syntax’ subscale. Consistent with our results, a recent review by Lai and Szatmari (2020) suggest that females with ASD may show higher linguistic abilities, mirroring normative sex differences and placing them closer to typically developing peers and away from males with ASD. However, these linguistic strengths may mask their real struggles with social communication, and complicate or delay the detection of their ASD symptoms (Parish-Morris et al. 2017; Lai and Szatmari 2020). The presence of early language delay has been related to earlier diagnosis of ASD (Goodwin et al. 2017; Lord et al. 2018). Early language delay was rare among females in the present sample, whose mean age at ASD diagnosis was higher compared with males. Although our findings may not seem surprising, they contrast with several studies that did not find significant sex differences in language and communication among ASD individuals (Tillmann et al. 2018; Solomon et al. 2012; Lawson et al. 2018). Due to the limited number of female participants in our study (*n* = 34), it is not possible to draw firm conclusions on potential sex differences. However, two large studies recently reported that children with ASD and more advanced language abilities, particularly females, were diagnosed later than non-verbal and minimally verbal children (McCormick et al. 2020; Salomone et al. 2016).

### Strengths and Limitations

A major strength of our study is the dimensional approach which enabled us to study language skills in a sample of children assessed for ASD with and without ASD diagnoses, increasing generalizability to the broader population of children evaluated for ASD. The large sample size compared to previous studies of CCC-2 in children with ASD, including a relatively large number of females, is another strength. Further, available data on age at inclusion and cognitive abilities allowed adjustment for these potential confounding factors.

Limitations include a potential selection bias, as referral for assessment in the present sample was based on concern. The participants may not be representative for children with autistic symptoms in the general population. Further, we relied upon parent report of structural and pragmatic language skills in everyday contexts as measured by the Norwegian version of the CCC-2. As this checklist is only suitable for verbal children who speak Norwegian, our results may be less applicable to younger children, children with no verbal language, as well as other languages. A small number of children with a *history* of hearing impairment were not excluded, as they were considered verbal and had completed the CCC-2. We used retrospective parent report on early language delay collected at inclusion (age from 4 to 18 years), introducing the possibility of recall bias. This information, however, was supplementary to available information in the child’s medical record. Although the precision of information regarding attainment of phrase speech at 2 years’ age may have varied, we do not consider it likely to have biased our results systematically. Further, the proportion of children with early language delay observed in the present study is comparable to a previous Norwegian study by Suren et al. (2019b). Mild or moderate deficits in social and communicative competence may be missed in the context of co-occurring difficulties, such as ADHD (Skuse et al. 2009), a common NDD in the present sample. As the proportion of individuals diagnosed with ADHD did not differ between the two diagnostic groups, we do not consider their inclusion to have biased our results in one direction. The large proportion with co-occurring ADHD, however, may have contributed to the observed late age at ASD diagnosis (11.5 years). Finally, the use of clinical diagnoses obtained from different clinics is a potential source of bias. Misclassification in both directions for ASD and the non-ASD disorders are considered possible, but not very probable. A recent review of patient records show that 95% of ASD diagnoses provided a high standard of documentation within the Norwegian specialist health service and meet the diagnostic criteria (Suren et al. 2019a).

### Clinical Implications

Language and communication skills are critical to the cognitive and social development of children, and highly predictive of academic and employment outcomes, regardless of the primary diagnosis (Norbury and Paul 2018; Conti-Ramsden and Durkin 2015). Children evaluated for suspected ASD commonly present with structural as well as pragmatic language impairments, that are likely to persist and to require on-going support as the child gets older. These impairments represent an important target of intervention. In a clinical setting, such interventions should be centered on the child’s age and profile of strength and needs, rather than the diagnostic category alone. They should be multifaceted, incorporating techniques for improving structural language skills, social communication and interaction, as well as using linguistic context to improve comprehension (Norbury 2014). Our results suggest that both language milestones and the CCC-2 may be helpful for identifying children with increased risk for structural language impairments, which needs to be managed separately from the presenting ASD symptoms.

## Conclusion

We found a large extent of structural as well as pragmatic language deficits in children evaluated for suspected ASD. Structural language deficits were associated with reduced pragmatic competence across the whole sample and more common among children with early language delay, while pragmatic language impairments were most profound in children with diagnosed ASD. Our results support the notion of pragmatic language impairment as a dimensional symptom profile potentially linked to several developmental risk factors, among them structural language deficits. This underscores the importance of including language skills assessment in the diagnostic evaluation of children with suspected ASD. Applied both in clinical and research settings language milestones have the potential for identifying a subgroup of children with increased risk for structural language impairments. These children may benefit from specific language interventions in addition to management of the core ASD symptoms.

## Supplementary Material and Sensitivity Analyses

For the purpose of comparison with previous samples we have included information on participant characteristics by ASD group status (Table

**Table 5:** Participant characteristics by diagnostic group (*N* = 177)

	ASD (*n* = 148)			Non-ASD (*n* = 29)		
	*n*	(%)	Mean (SD)	*n*	(%)	Mean (SD)
Male sex	119	80.4		24	82.8	
Age (years) at inclusion	148		12.5 (3.2)	29		11.0 (3.7)
Age (years) at ASD diagnosis	144		11.5 (3.3)			
Autistic symptom severity						
ADI-R nonverbal total	66		22.2 (9.5)	10		10.0 (10.6)
SCQ total	94		15.8 (7.5)	20		10.3 (8.2)
SRS raw total	136		86.7 (27.7)	26		66.2 (34.8)
Age (years) at cognitive testing	142		10.0 (3.3)	26		10.0 (3.7)
Nonverbal IQ	137		102.7(18.4)	24		101.6(19.0)
Verbal IQ	138		90.8 (17.0)	25		94.8 (15.9)
Early language milestones						
One word 1 year (no)	30	24.0		2	10.5	
Two words 2 year (no)	31	25.6		7	36.8	
Diagnoses						
Intellectual disability (F70-79)	7	4.8		1	3.4	
ADHD (F90)	86	59.3		17	63.0	
Communication disorder (F80)	5	3.4		2	7.4	
Specific learning disorder (F81 + F83)	12	8.3		6	22.2	
Motor disorders (F82 + F95)	22	15.2		5	18.5	
Epilepsy	8	5.4		2	6.9	
Cerebral palsy	1	0.7		1	3.6	
Other NDD (F94)	1	0.7		0	0	
Motor disorders (F82 + F95)	22	15.2		5	18.5	
Epilepsy	8	5.4		2	6.9	
Cerebral Palsy	1	0.7		1	3.6	
Other NDD (F94)	1	0.7		0	0	
No of NDDs						
0	0			5	19.2	
1	47	32.9		11	42.3	
≥ 2	96	67.1		10	38.5	
Prematurity (yes)	19	14.3		7	25.9	
Paternal age (years)	97		32.3 (5.9)	21		33.2 (6.3)
Maternal age (years)	106		29.8 (5.2)	25		29.4 (5.3)
Ethnicity						
European (Caucasian)	129	89.0		28	100.0	

Data are expressed as *n* (%) or mean (SD). The denominator for the reported proportions in this table excludes those with missing data. IQ was obtained from various age-appropriate standardized tests

*ADI-R* Autism Diagnostic Interview-Revised, *ASD* autism spectrum disorder, *SCQ* Social Communication Questionnaire, *SRS* Social Responsiveness Scale, *NDD* neurodevelopmental disorder

5). The Norwegian CCC-2 manual (Bishop 2011, p. 72) provides a description on how to assess the internal consistency of the parents’ answers. In cases of invalid consistency check, it is recommended not to interpret the individuals’ test result. In the present sample, we compared participant characteristics between individuals with valid consistency check on the CCC-2 (*n* = 153) and *n* = 22 individuals with invalid CCC-2 scores not passing the instruments’ consistency check (Table

**Table 6:** Participant characteristics by CCC-2 consistency check (*n* = 175)

	Valid (*n* = 153)			Not valid (*n* = 22)		
	*n*	(%)	Mean (SD)	*n*	(%)	Mean (SD)
Male sex	123	80.4		18	81.8	
ASD	127	83.0		20	90.9	
Age (years) at inclusion	153		12.3 (3.4)	22		12.3 (2.7)
Age (years) at ASD diagnosis	123		11.4 (3.4)	20		11.6 (2.7)
Nonverbal IQ	138		102.8 (18.8)	21		100.2 (15.3)
No of NDDs						
0	4	2.7		0	0	
1	56	38.1		2	10.0	
≥ 2	87	59.2		18	90.0	
Early language development						
One word 1 year (no)	26	21.0		6	31.6	
Two words 2 year (no)	33	27.5		5	26.3	
CCC-2 composite scores						
GCC (sum scales A–H)	153		40.2 (18.3)	22		30.0 (9.8)
Structural Language Score (sum scales A–D)	153		22.7 (10.4)	22		15.6 (6.3)
General Pragmatics Score (sum scales E–H)	153		17.5 (10.1)	22		14.3 (4.9)
Ethnicity						
European (Caucasian)	135	90.0		21	95.5	

Data are expressed as *n* (%) or mean (SD). The denominator for the reported proportions in this table excludes those with missing data. IQ was obtained from various age-appropriate standardized tests. 2 participants had missing information on results of the consistency check

*ASD* autism spectrum disorder, *CCC-2* Children’s Communication Checklist Second Edition, *NDD* neurodevelopmental disorder

6). Participant characteristics did not differ substantially between these two groups, except that a larger proportion of children not passing the consistency check were diagnosed with two or more neurodevelopmental disorders (*p* = .007). Children with invalid consistency check, however, had lower scores on the General Communication and Structural Language composites, indicating larger impairment in general communication and structural language skills. Further, the group with invalid consistency check also had lower pragmatic scores, that were more proportionate to their structural language skills. In the present study, analyses with and without exclusion of individuals with invalid consistency check did not affect the main outcomes substantially. The proportion of parents (*n* = 22/175; 12.6%) that were inconsistent in their answers on the CCC-2 in the present study is in line with findings by Geurts and Embrechts (2008) (9.3–22.8%), and most likely due to the change in questions types throughout the CCC-2. During the first part of the CCC-2 questions focusing on difficulties are negatively formulated, whereas the last 20 questions focusing on strengths are positively formulated. Although instructions clearly state that there is a change in question type, answers to the last questions may be given as if they were still negatively formulated. Consequently, the scaled scores of each subscale will be higher (indicating less difficulties) than if the questions were answered consistently, underestimating the difficulties a child encounter. Considering results on the consistency check is important when using the CCC-2 in individual assessment of the communication pattern of a child in a clinical setting, where an invalid consistency check should elicit careful consideration of possible reasons for the invalid result. However, our results indicate that not passing the reliability check may not be a random event, and that exclusion of these individuals may bias results on a group level and underestimate the true extent of structural language deficits in research samples.

In order to assess the potential impact of including children with intellectual disability (*n* = 8) on CCC-2 composite scores in the present study, as well as estimates of group differences and associations between structural and pragmatic language skills, we checked whether these children represented outliers in the distribution of CCC-2 scores (Fig. 

Fig. 4 Distribution of Structural Language and General Pragmatics composite scores, in the group with (*n* = 8) and without (*n* = 169) co-occurrent intellectual disability

4). Further, main analyses were repeated with these individuals excluded, resulting in a modest attenuation of the results, not affecting the statistical significance of our findings.

In the present study we have chosen to present scaled scores from the CCC-2 as recommended in the CCC-2 manual. Further, we have chosen to use the Structural Language and the General Pragmatics composite scores, although not described in the manual. No Norwegian norms are available for these composite scores. However, since 10 is the average of the scaled scores on each of the four subscales for both indexes, a putative mean value of 40 is expected for each. Previous studies reporting these composites have presented their results as raw totals (Kuijper et al. 2017; Baixauli-Fortea et al. 2019), while we have chosen to report scaled scores. We therefore present some of our results as CCC-2 raw scores for comparison (Fig. 

Fig. 5 Distribution of Structural Language and General Pragmatics composite scores (raw scores) across the study sample, and their linear associations in the group with and without diagnosed autism spectrum disorder (ASD; *n* = 147 and non-ASD; *n* = 28)

5 and Table

**Table 7:** CCC-2 raw scores (a low score indicates better language ability): means, standard deviations, and comparisons between diagnostic groups

	Groups				Difference		
	ASD		Non-ASD				
	*n* = 147		*n* = 28				
	Mean	SD	Mean	SD	Estimate	95% CI	*p*
CCC-2 subscale scores							
A. Speech	2.4	3.2	3.5	4.1	− 1.2	(− 2.5 to .2)	.09
B. Syntax	2.3	2.7	2.8	3.1	− .5	(− 1.6 to .6)	.36
C. Semantics	5.7	3.4	5.5	4.4	.1	(− 1.3 to 1.6)	.85
D. Coherence	6.8	4.3	6.6	4.1	.2	(− 1.5 to 2.0)	.79
E. Inappropriate initiation	8.5	4.6	8.3	5.4	.3	(− 1.7 to 2.2)	.79
F. Stereotyped language	4.4	3.4	3.6	3.0	.8	(− .6 to 2.1)	.28
G. Use of context	7.3	4.4	6.9	4.3	.4	(− 1.3 to 2.2)	.63
H. Nonverbal communication	7.7	4.5	5.9	4.0	1.8	(.9 to − .005)	.05
I. Social relations	7.6	4.0	5.7	4.2	2.0	(.3 to 3.6)	.02
J. Interests	9.6	4.6	7.3	3.8	2.3	(.5 to 4.1)	.01
CCC-2 composite scores							
Structural Language Score (sum scales A–D)	17.1	10.7	18.4	13.2	− 1.3	(− 5.9 to 3.2)	.57
General Pragmatics Score (sum scales E–H)	27.9	14.3	24.7	14.3	3.2	(− 2.6 to 9.1)	.28

*ASD* autism spectrum disorder, *CCC-2* Children’s Communication Checklist Second Edition, *SD* = standard deviation, *CI* confidence interval, *p p*-value for independent samples t-test

7). Kuijper et al. (2017) reported a mean (SD) Structural Language Score in the ASD group of 20.4 (9.0), and a mean (SD) General Pragmatic Score of 37.4 (13.1), both of which are higher (indicating larger deficits) compared with the present sample. In a more recent study, Baixauli-Fortea et al. (2019) report a mean (SD) Structural Language Score in the ASD group of 19.0 (9.4), which is close to the observed value in the present sample. There are, however, important differences between these two and the present study; smaller sample sizes (*n* = 36 and *n* = 52), the inclusion of only participants with normal range cognitive abilities, as well as a more limited age range under study (6–12 and 7–11 years), which may limit comparability.
