# Supplementary figures and images for: Structural and Pragmatic Language Impairments in Children Evaluated for Autism Spectrum Disorder (ASD)
Source: J Autism Dev Disord. 2021 Jan 30;53(2):701–19. doi: 10.1007/s10803-020-04853-1 (PMC9944009; doi:10.1007/s10803-020-04853-1)

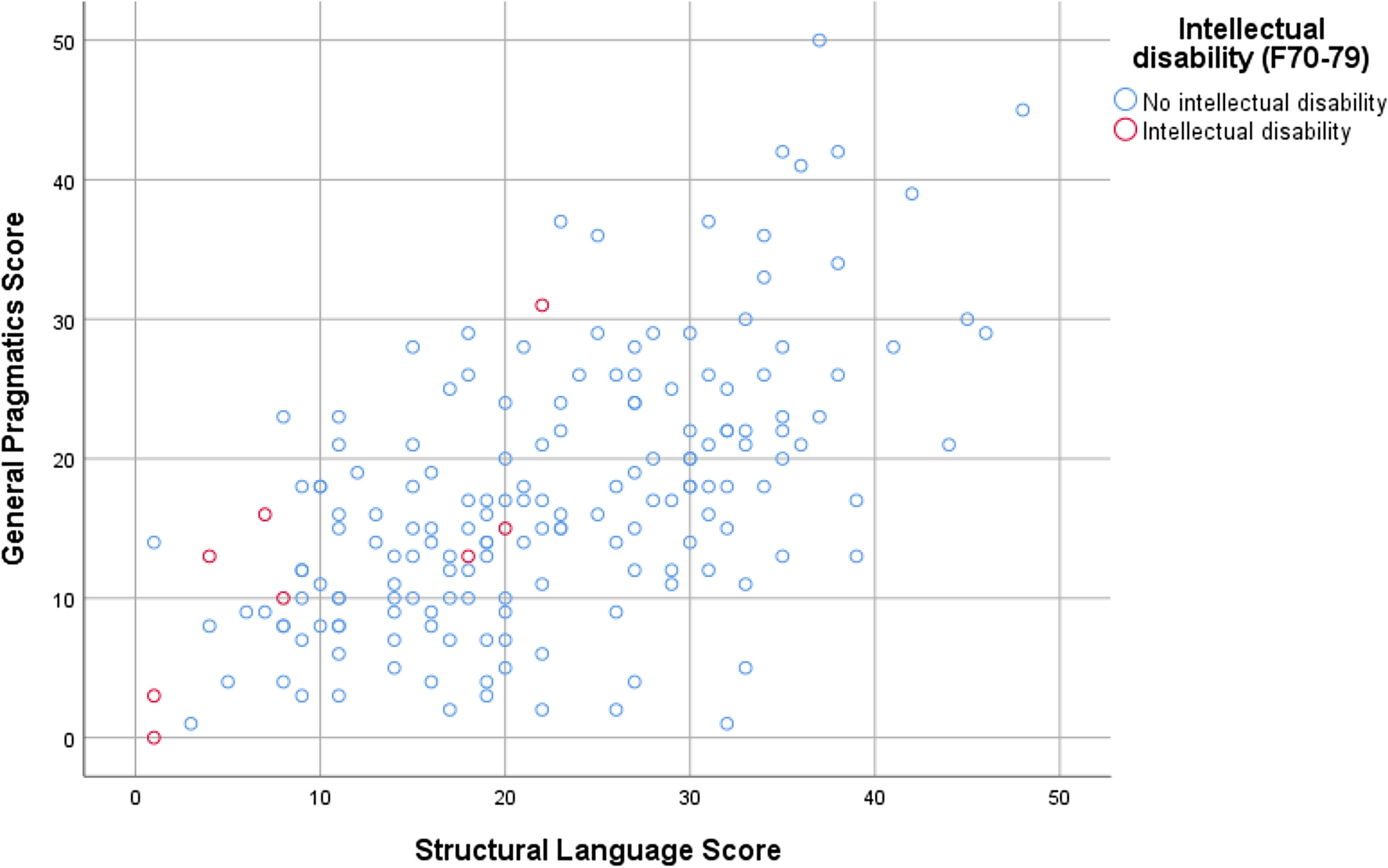

Supplement: Supplementary file 1 — Distribution of Structural Language and General Pragmatics composite scores, in the group with (n = 8) and without (n = 169) co-occurrent intellectual disability [file 10803_2020_4853_Fig4_HTML.png]

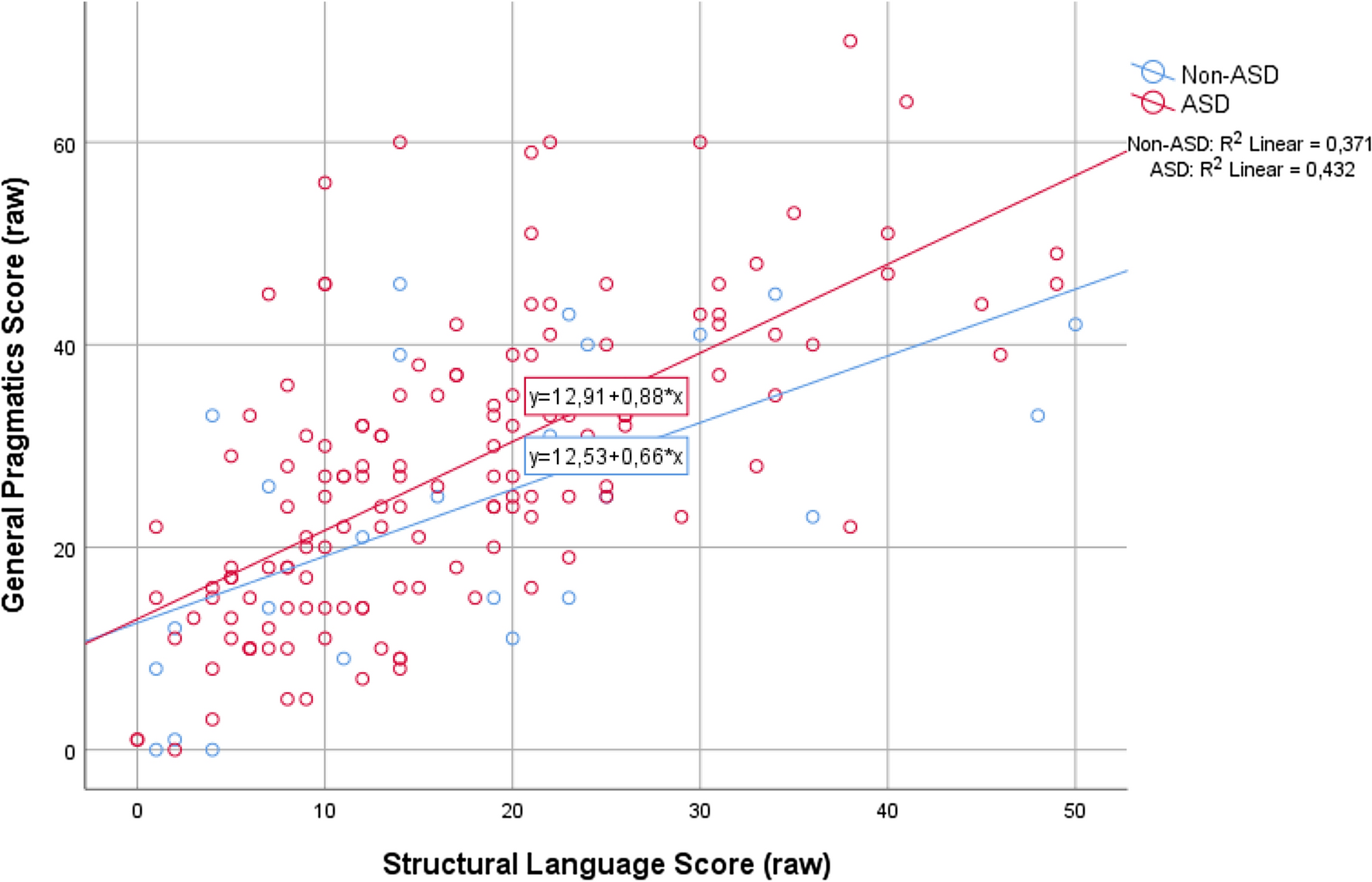

Supplement: Supplementary file 2 — Distribution of Structural Language and General Pragmatics composite scores (raw scores) across the study sample, and their linear associations in the group with and without diagnosed autism spectrum disorder (ASD; n = 147 and non-ASD; n = 28) [file 10803_2020_4853_Fig5_HTML.png]
